# Sale of Tea in Russia

**Published:** 1891-07

**Authors:** 


### SALE OF TEA IN RUSSIA.

We learn, says the London *Grocer*, that to insure the public against the possibility of purchasing adulterated and spurious tea, tea dealers in Russia are allowed, since May, 1889, to sell their wares under government labels, which are placed on packets of tea of various weights, by persons employed by the government for that purpose, and who work under the control of official inspectors. The cost of labeling which is small, is defrayed out of the money realized by the sale of the labels. The labeling is not imperative, but most of the tea merchants in the retail trade have recourse to this expedient to increase their sales. The adoption of some measure of this kind, it is stated, was necessary in order to restore the confidence of the public at large in the genuineness of the tea offered for sale. To some extent this labeling has checked the traffic in adulterated teas in the two capitals. In the provincial towns adulteration of tea and other necessaries of life continues to be largely practiced.

